# Biologics in Orthopaedics: Is Platelet-Rich Plasma Ready for Widespread Clinical Use?

**DOI:** 10.7759/cureus.90528

**Published:** 2025-08-19

**Authors:** Anirudh Dwajan

**Affiliations:** 1 Orthopaedics, Jawaharlal Institute of Postgraduate Medical Education and Research, Pondicherry, IND

**Keywords:** back pain, biological therapy, biologics, musculoskeletal disorders, orthopaedics, osteoarthritis, platelet-rich plasma, regenerative medicine, spine, tendinopathy

## Abstract

Biological therapies such as platelet-rich plasma (PRP), stem cells, and bone marrow aspirate concentrate have gained attention in orthopaedics as potential alternatives to conventional interventions. PRP, in particular, has been extensively studied in spinal disorders, tendinopathies, and cartilage lesions. While encouraging evidence suggests symptomatic improvement and functional recovery in selected patients, limitations such as a lack of standardisation, variability in preparation, and limited long-term outcomes hinder universal adoption. In our clinical experience, patients are increasingly curious about PRP, often asking about its cost, availability, and expected outcomes. Although many report short-term relief, sustained long-term benefits remain inconsistent. This editorial discusses the current evidence, practical challenges, and future direction of PRP in orthopaedic practice.

## Editorial

Introduction

The pursuit of biological solutions in orthopaedics has accelerated over the past two decades. Among various regenerative therapies, platelet-rich plasma (PRP) has emerged as the most extensively studied and clinically applied option. Derived from autologous blood through centrifugation, PRP contains concentrated platelets, growth factors, and cytokines that may modulate inflammation and stimulate tissue repair [1]. Its appeal lies in being minimally invasive, relatively cost-effective, and autologous in origin. Nevertheless, its efficacy, reproducibility, and long-term clinical value remain debated. This article summarises current evidence and highlights priorities for its future application.

Applications in orthopaedics

In spinal disorders, intradiscal PRP injections have shown promise in the management of chronic lumbar discogenic pain. A randomised controlled trial demonstrated that PRP injections resulted in significant improvements in pain and function compared with saline, suggesting that PRP may enhance extracellular matrix repair and reduce inflammation in intervertebral discs [2]. Clinically, patients with chronic low back pain increasingly view PRP as an alternative to surgery, reflecting rising awareness of biological therapies.

Tendinopathies represent another major field of PRP application, particularly in lateral epicondylitis. A large multicenter randomised controlled trial reported superior pain relief and functional improvement in patients receiving PRP compared with placebo, reinforcing its potential role in chronic tendon injuries [3]. Despite this, heterogeneity in outcomes remains, influenced by disease chronicity, technique of preparation, and injection protocol. Younger athletes, in particular, appear receptive to PRP, often preferring it over corticosteroid injections due to its biological appeal.

Cartilage pathology and osteoarthritis have also been extensively investigated. A systematic review and meta-analysis found that intra-articular PRP injections produced greater improvements in pain and function than hyaluronic acid, particularly in knee osteoarthritis [4]. Beyond symptom control, PRP is being studied as an adjunct to cartilage repair techniques such as microfracture and autologous chondrocyte implantation, with the aim of enhancing tissue integration and long-term outcomes.

Limitations and challenges

Despite promising evidence, several limitations restrict the widespread use of PRP in orthopaedics. One of the greatest challenges is the lack of standardisation. Numerous commercial preparation systems exist, each producing variable concentrations of platelets, leucocytes, and growth factors, leading to inconsistencies in both research and clinical practice [5]. Evidence quality also remains a concern, as many clinical studies include small cohorts, methodological weaknesses, and short follow-up periods. In addition, outcomes vary across different conditions and patient groups, making it difficult to define clear indications. From a practical perspective, clinicians face challenges in counselling patients, as many approach PRP with expectations of a definitive cure, necessitating a realistic discussion about its experimental nature and inconsistent results.

Future directions

For PRP to transition into mainstream orthopaedic care, several priorities must be addressed. Standardised preparation techniques and classification systems are necessary to enable meaningful comparisons between studies [5]. Large multicenter randomised controlled trials with long-term follow-up are required to establish durability of benefits and identify responders more reliably. Advances in biomarker research may also help refine patient selection and optimise outcomes. Finally, the development of registries to capture real-world data will strengthen safety monitoring and enhance clinical applicability. Achieving these goals will require close collaboration between clinicians, researchers, and industry to ensure reproducibility and clinical relevance.

Conclusion

PRP represents an exciting but incompletely defined tool in orthopaedics. Its potential to improve outcomes in spinal disorders, tendinopathies, and osteoarthritis is supported by encouraging evidence, yet the absence of standardisation and long-term data continues to limit its universal adoption. At present, PRP should be regarded as an adjunctive option rather than a replacement for established therapies. Careful patient selection, transparent counselling, and high-quality research remain key to defining its ultimate role in clinical practice.

Learning point

PRP offers therapeutic potential across spinal disorders, tendinopathies, and osteoarthritis, but widespread adoption depends on standardisation, robust clinical trials, and long-term evidence.
